# Endothelin-1 Upregulates Activin Receptor-Like Kinase-1 Expression via G*i*/RhoA/Sp-1/Rho Kinase Pathways in Human Pulmonary Arterial Endothelial Cells

**DOI:** 10.3389/fcvm.2021.648981

**Published:** 2021-02-23

**Authors:** Koichi Sugimoto, Tetsuro Yokokawa, Tomofumi Misaka, Takashi Kaneshiro, Shinya Yamada, Akiomi Yoshihisa, Kazuhiko Nakazato, Yasuchika Takeishi

**Affiliations:** ^1^Department of Cardiovascular Medicine, Fukushima Medical University, Fukushima, Japan; ^2^Department of Pulmonary Hypertension, Fukushima Medical University, Fukushima, Japan

**Keywords:** pulmonary hypertension, small GTP protein, endothelin, ACVRL-1, pulmonary endothelial cells

## Abstract

**Background:** Pulmonary arterial hypertension (PAH) is characterized by pulmonary vasoconstriction and organic stenosis. It has been demonstrated that endothelin-1 (ET-1) induces pulmonary vasoconstriction through the activation of RhoA. In addition, a gene mutation of activin receptor-like kinase (ACVRL)-1 is recognized in PAH patients. However, little is known about the association between ET-1 and ACVRL-1.

**Objective:** In the present study, we aimed to investigate the effect of ET-1 on ACVRL-1 expression and delineate the involvement of the G*i*/RhoA/Rho kinase pathway.

**Methods:** ET-1 was added to culture medium of human pulmonary arterial endothelial cells (PAECs). Pre-treatment with pertussis toxin (PTX) or exoenzyme C3 transferase (C3T) was performed for inhibition of G*i* or RhoA, respectively. Rho kinase was inhibited by Y27632. Mithramycin A was used for inhibition of Sp-1, which is a transcriptional factor of ACVRL-1. The active form of RhoA (GTP-RhoA) was assessed by pull-down assay.

**Results:** ACVRL-1 expression was increased by ET-1 in the PAECs. Pull-down assay revealed that ET-1 induced GTP-loading of RhoA, which was suppressed by pre-treatment with PTX or C3T. Further, PTX, C3T, and Y27632 suppressed the ET-1-induced ACVRL-1 expression. ET-1 increased the activity of the ACVRL-1 promoter and stabilized the ACVRL-1 mRNA. Sp-1 peaked 15 min after adding ET-1 to the PAECs. PTX and C3T prevented the increase of Sp-1 induced by ET-1. Inhibition of Sp-1 by mithramycin A suppressed ET-1-induced ACVRL-1 upregulation.

**Conclusion:** The present study demonstrated that ET-1 increases ACVRL-1 expression in human PAECs *via* the G*i*/RhoA/Rho kinase pathway with the involvement of Sp-1.

## Introduction

Pulmonary arterial hypertension (PAH) is a disease with poor prognosis that is characterized by pulmonary vasoconstriction and organic stenosis due to abnormal proliferation of pulmonary artery endothelial cells and smooth muscle cells (1–3). It is considered that endothelial dysfunction is associated with these vascular pathologies (4, 5); however, their detailed molecular mechanisms are still unknown.

Endothelin-1 (ET-1) is a major vasoconstrictor derived from endothelial cells (6). Today, endothelin receptor agonist is widely used for PAH treatment and has contributed to the improvement of PAH prognosis. This shows that endothelin plays a crucial role in PAH. It has been demonstrated that ET-1 induces pulmonary vasoconstriction through the activation of RhoA, which is a small GTP protein (7). Many studies have reported that the RhoA/Rho-kinase pathways are implicated in pulmonary hypertension (8–10). Additionally, endothelin receptors are G-protein-coupled receptors (7), and we have previously demonstrated that G*i*, which is a heterotrimeric G protein, functions upstream of RhoA activation (11).

Activin receptor-like kinase-1 (ACVRL-1) is one of the type I cell surface receptors for the transforming growth factor-β (TGF-β) family that is mainly expressed in vascular endothelial cells (12). A gene mutation of ACVRL-1 is recognized in idiopathic or heritable PAH patients (13). Although both ET-1 and ACVRL-1 are important molecules for the pathogenesis of PAH, little is known about the association between them.

In the present study, we aimed to investigate the effect of ET-1 on ACVRL-1 expression and delineate the involvement of the Gi/RhoA/Rho kinase pathway in pulmonary arterial endothelial cells.

## Methods

### Materials

Most of the reagents used in this study have been described previously (14–16). Recombinant human ET-1 was obtained from R&D systems (Minneapolis, MN, USA), and cell permeable exoenzyme C3 transferase (C3T) was purchased from Cytoskeleton, Inc. (Denver, CO, USA). Pertussis toxin (PTX), actinomycin D, and mithramycin A were purchased from Merck KGaA (Darmstadt, Germany). Y27632 was purchased from the FUJIFILM Wako Pure Chemical Corporation (Osaka, Japan).

### Preparation of Endothelial Cells

Human pulmonary arterial endothelial cells (PAECs) were purchased from PromoCell (Heidelberg, Germany). PAECs were cultured in endothelial medium (EBM-2, PromoCell, C-22211) supplemented with 2% fetal calf serum (FCS) and epidermal growth factor (5 ng/ml), basic fibroblast growth factor (10 ng/ml), insulin-like growth factor (20 ng/ml), vascular endothelial growth factor 165 (0.5 ng/ml), ascorbic acid (1 μg/ml), heparin (22.5 μg/ml), and hydrocortisone (0.2 μg/ml) (all from PromoCell). Confluent PAECs from passage 5 through 10 in 2% FCS containing medium were used for all experiments.

### Western Blotting

Western blotting was performed as described previously (14–16). The lysates of lung tissues were mixed at a ratio of 4:1 with loading buffer [75 mM Tris-HCl, pH 6.8; 10% glycerol; 3% 2-mercaptoethanol, and 2% sodium dodecyl sulfate (SDS)] and heated at 95°C for 10 min. Aliquots containing 20 μg of protein were subjected to SDS-polyacrylamide gel electrophoresis, and the proteins were then transferred onto polyvinylidene difluoride membranes (Merck KGaA). After incubation with blocking solution at room temperature for 30 min, the membranes were incubated for 1 h at room temperature with a monoclonal antibody to RhoA (Santa Cruz Biotechnology, Santa Cruz, CA) and ACVRL-1 (Abcam, Cambridge, UK) at a dilution of 1:500, and to β-actin (Santa Cruz Biotechnology) diluted 1:1,000 or a rabbit polyclonal antibody to Sp-1 (GeneTex, Inc., Irvine, CA, USA) diluted 1:1,000 for immunoblotting. The signals from immunoreactive bands were visualized by a Clarity™ Western ECL Substrate (Bio-Rad Laboratories, Inc., Hercules, CA, USA). The optical densities of individual bands were analyzed using Image J 1.48.

### GTP/GDP Exchange of RhoA

GTP-bound active forms of RhoA was assessed using a commercially available assay kit (Cytoskeleton Inc.) according to the manufacturer's instructions (17).

### Quantitative Reverse Transcription Polymerase Chain Reaction

Total RNA was extracted from PAECs using TRIzol reagent (Invitrogen Carlsbad, CA, USA) and reverse transcribed into first-strand cDNA with a ReverseTra Ace qPCR RT kit (Toyobo Co., Ltd., Osaka, Japan). The cDNA was subjected to quantitative polymerase chain reaction (qPCR) using a Thunderbird SYBR qPCR Mix (Toyobo) in a CFX Connect Real-Time PCR Detection System (Bio-Rad Laboratories, Inc.). Glyceraldehyde-3-phosphate dehydrogenase (GAPDH) was used as an internal control. Primers were designed on the basis of GenBank sequences (ACVRL-1, NM_009612.3 and GAPDH, NM_001289726.1). qPCR was run in duplicates, and the delta-delta Ct method was applied for quantification.

### DNA Transfection and Luciferase Assay

We cloned a firefly luciferase reporter construct ACVRL-1/Luc containing the human putative promoter region of ACVRL-1 (GeneBank: NC_000012.12, position 51906383 to 51907627) into the pGL3-Basic vector (18). pNL1.1.TK (NLuc/TK) was used as a control vector. The constructs were co-transfected into 70% confluent PAECs using a Screenfect A (FUJIFILM Wako Pure Chemical Corporation). Forty-eight hours after transfection, cell lysates were assayed for luciferase activity using a Nano-Glo Dual Luciferase Reporter Assay System (Promega Corporation, Madison, WI, USA) in accordance with the manufacturer's instructions. Cell culture experiments were performed in triplicate.

### Determination of ACVRL-1 mRNA Stability

To analyze the effect of ET-1 on ACVRL-1 mRNA stability, PAECs were stimulated by ET-1 for 6 h with or without Y27632, then 5 μg/mL of actinomycin D was added. ACVRL-1 mRNA levels were determined by qPCR.

### Statistical Analysis

Statistical analyses were performed using ANOVA with Tukey's *post-hoc*-test or Student's *t*-test where appropriate. A value of *P* < 0.05 was considered significant. Data are expressed as means ± standard errors (SE).

## Results

### Effects of ET-1 on Expression of ACVRL-1 in PAECs

**Figure 1:**
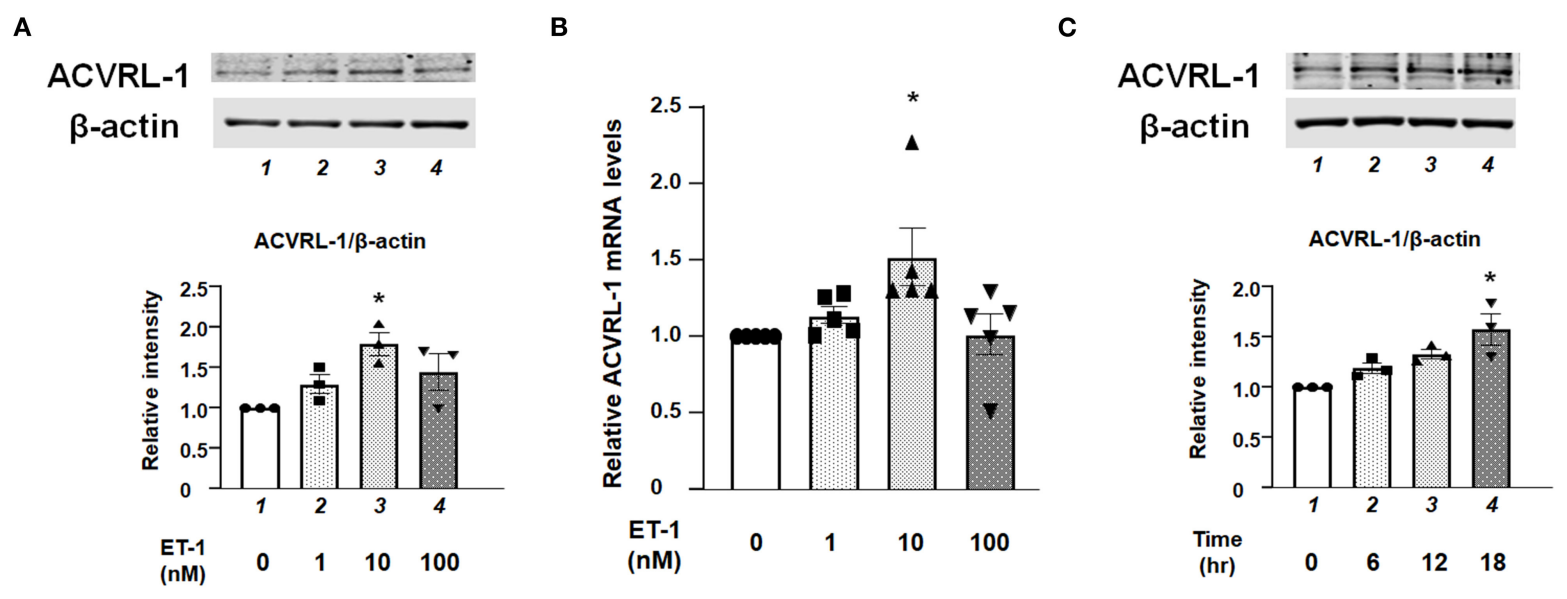
**(A)** Effects of ET-1 on expression of ACVRL-1 in human PAECs as determined by Western blotting. Lane 1, PAECs alone (control); lane 2, PAECs + ET-1 (1 nmol/L); lane 3, PAECs + ET-1 (10 nmol/L); and lane 4, PAECs + ET-1 (100 nmol/L). PAECs were stimulated by ET-1 at the indicated concentration for 18 h. Quantitative results of ACVRL-1 were normalized by the levels of β-actin. Representative immunoblots are shown at the top. **(B)** Effects of ET-1 on RNA expression of ACVRL-1 in human PAECs as determined by qPCR. Lane 1, PAECs alone (control); lane 2, PAECs + ET-1 (1 nmol/L); lane 3, PAECs + ET-1 (10 nmol/L); and lane 4, PAECs + ET-1 (100 nmol/L). PAECs were stimulated by ET-1 at the indicated concentration for 6 h. Quantitative results of ACVRL-1 were normalized by the levels of GAPDH. **(C)** Time course of ACVRL-1 expression in PAECs caused by ET-1 (10 nmol/L) stimulation as determined by Western blotting. Lane 1, PAECs alone (control); lane 2, PAECs + ET-1 (6 h); lane 3, PAECs + ET-1 (12 h); and lane 4, PAECs + ET-1 (18 h). Quantitative results of ACVRL-1 were normalized by the levels of β-actin. Representative immunoblots are shown at the top. Bars are means ± SE of three to five separate experiments. *P < 0.05 vs. control.

Western blotting and qPCR demonstrated that protein and mRNA expressions of ACVRL-1 were increased by ET-1 stimulation in the PAECs (Figures 1A,B). Further, these results indicate that the upregulation of ACVRL-1 was not necessarily dependent on the dose of ET-1. Figure 1C shows the time course of ACVRL-1 expressions in response to ET-1 stimulation.

### Levels of GTP-Bound Active Form of RhoA in ET-1-Stimulated PAECs

**Figure 2:**
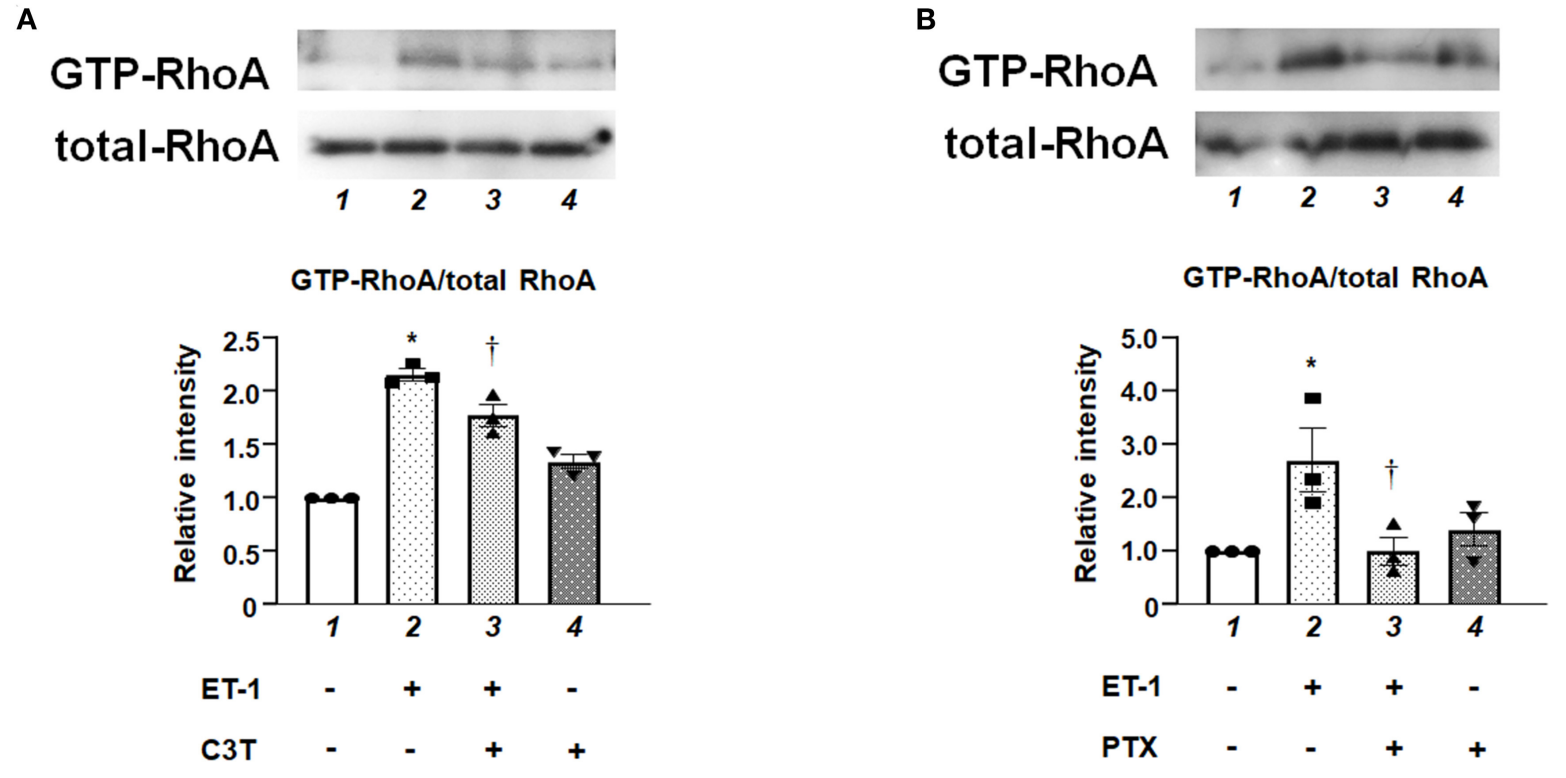
**(A)** Levels of GTP-bound active form of RhoA in cultured human PAECs. PAECs were pre-treated with C3T (0.25 μg/mL) overnight before adding ET-1. After 5 min of ET-1 stimulation (10 nmol/L), GTP-loading of RhoA was determined by pull-down assays. Lane 1, PAECs alone (control); lane 2, PAECs + ET-1; lane 3, PAECs + ET-1 + C3T; and lane 4, PAECs + C3T. **(B)** PAECs were pretreated with 100 ng/mL of PTX overnight before adding ET-1. After 5 min of ET-1 stimulation (10 nmol/L), RhoA activation was determined by pull-down assay. Lane 1, PAECs alone (control); lane 2, PAECs + ET-1; lane 3, PAECs + ET-1 + PTX; and lane 4, PAECs + PTX. Quantitative results of GTP-RhoA were normalized by total RhoA levels. Bars are mean ± SE of quantitative densitometric analyses from three separate experiments. Representative immunoblots are shown at the top. *P < 0.05 vs. lane 1, ^†^*P* < 0.05 vs. lane 2.

Pull-down assay revealed that ET-1 rapidly induced a GTP-loading of RhoA, which is an active form of RhoA in the PAECs, whereas the levels of RhoA in whole cell lysates were not changed. C3T prevented ET-1-induced RhoA activation (Figure 2A). Figure 2B shows that the ET-1-induced RhoA activation was suppressed by pre-treatment with PTX, suggesting that G*i* is upstream of RhoA activation.

### Effect of Gi/RhoA/Rho Kinase Inhibition on ACVRL-1 Expression Induced by ET-1 in PAECs

**Figure 3:**
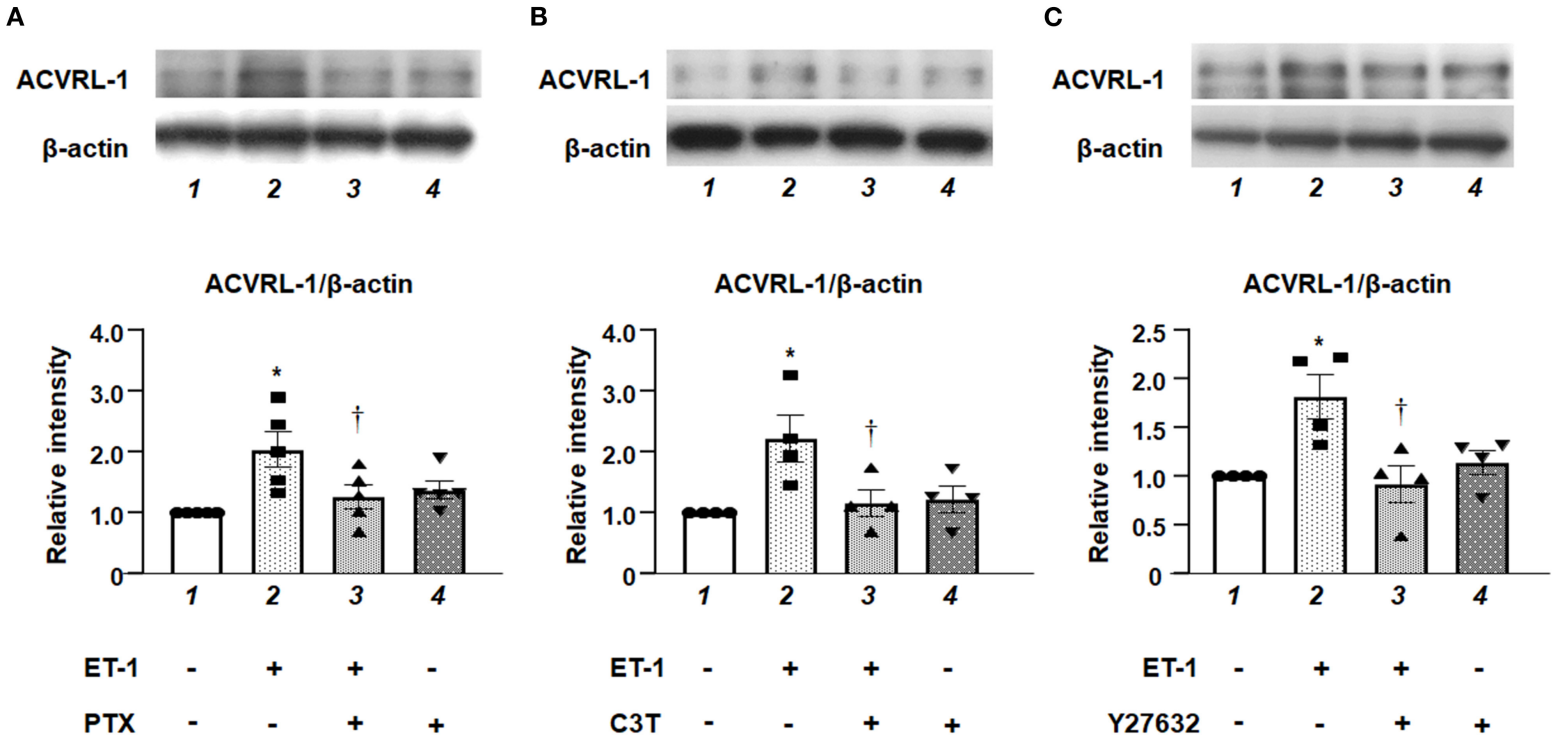
**(A)** Effect of G*i* inhibition by PTX on ACVRL-1 expression induced by ET-1 in PAECs, as determined by Western blotting. After overnight treatment with 100 ng/mL of PTX, ET-1 (10 nmol/L) was added to PAECs, and after 18 h, Western blotting of ACVRL-1 was performed. Lane 1, PAECs alone (control); lane 2, PAECs + ET-1; lane 3, PAECs + ET-1 + PTX; and lane 4, PAECs + PTX. **(B)** Effect of RhoA inhibition by C3T on ACVRL-1 expression induced by ET-1 in PAECs, as determined by western blotting. After overnight treatment with 0.25 μg/mL of C3T, ET-1 (10 nmol/L) was added to PAECs, and after 18 h, western blotting of ACVRL-1 was performed. Lane 1, PAECs alone (control); lane 2, PAECs + ET-1; lane 3, PAECs + ET-1 + C3T; and lane 4, PAECs + C3T. **(C)** Effect of Y-27632 on ACVRL-1 protein levels in PAECs. Ten μmol/L of Y-27632 were added to PAECs simultaneously with ET-1 stimulation (10 nmol/L), and ACVRL-1 protein levels were determined by western blotting after 18 h of culture. Lane 1, PAECs alone (control); lane 2, PAECs + ET-1; lane 3, PAECs + ET-1 + Y27632; and lane 4, PAECs + Y27632. Quantitative results of ACVRL-1 were normalized by the levels of β-actin. Data are expressed as mean ± SE of four to five experiments. Representative immunoblots are shown at the top. *P < 0.05 vs. lane 1, ^†^*P* < 0.05 vs. lane 2.

We examined the effects of inhibition of either G*i*, RhoA, or Rho kinase on the upregulation of ACVRL-1 expression in ET-1-stimulated PAECs. Western blotting showed that PTX (Figure 3A), C3T (Figure 3B), and Y27632 (Figure 3C) suppressed ET-1-induced ACVRL-1 expression. These results indicate that ET-1 increased the ACVRL-1 expression mediated *via* the G*i*/RhoA/Rho kinase signaling pathway.

### Promoter Activity and mRNA Stability of ACVRL-1 in ET-1-Stimilated PAECs

**Figure 4:**
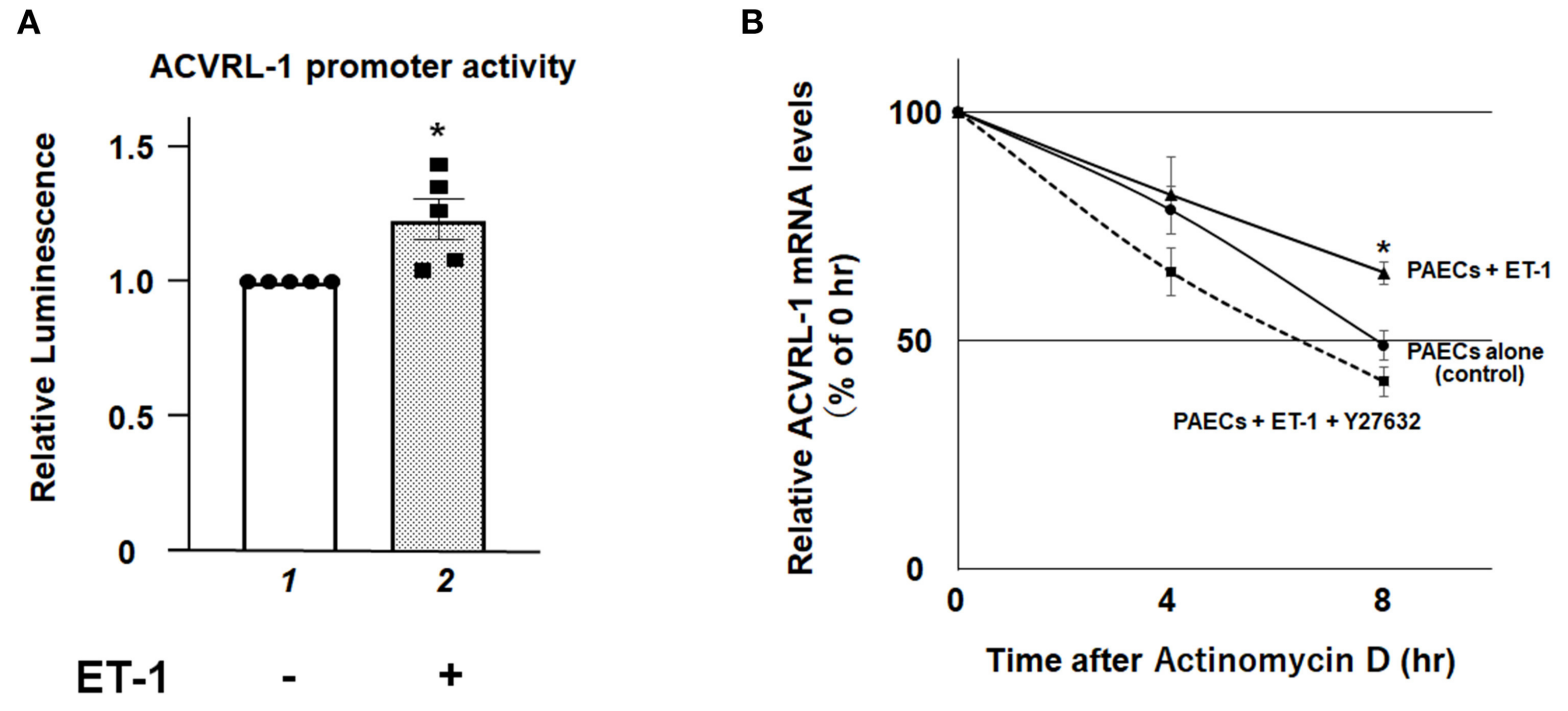
**(A)** The promoter activity of the ACVRL-1 gene in PAECs was determined after 6 h of incubation with or without ET-1 (10 nmol/L). Data are expressed as means ± SE of five separate experiments. *P < 0.05 vs. lane 1. **(B)** PAECs were stimulated by ET-1 (10 nmol/L) with or without Rho kinase inhibition by Y27632. After adding of 5 μg/mL of actinomycin D, ACVRL-1 mRNA levels were determined by qPCR at the indicated time points. Quantitative results of ACVRL-1 were normalized by the levels of GAPDH. Data are expressed as means ± SE of five separate experiments. *P < 0.05 vs. control, PAECs + ET-1 + Y27632.

To elucidate further mechanisms of ET-1-induced ACVRL-1 upregulation, we evaluated the promoter activity of ACVRL-1 and the stability of mRNA of ACVRL-1. Figure 4A shows that transcriptional activity of the ACVRL-1 promoter was increased by ET-1. In addition, ET-1 stabilized mRNA of ACVRL-1, and this stabilization was reversed to the control level by Rho kinase inhibition by Y27632 (Figure 4B).

### Effect of Gi/RhoA Inhibition on Sp-1 Induced by ET-1 in PAECs

**Figure 5:**
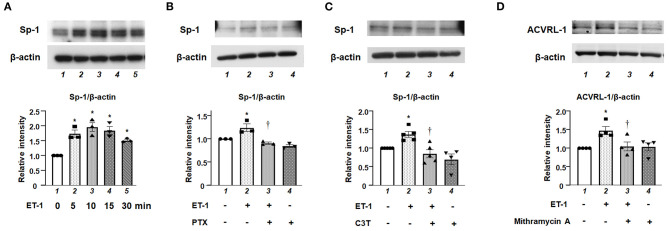
**(A)** Levels of Sp-1 protein as determined by Western blotting in cultured human PAECs 5 to 30 min after adding 10 nmol/L of ET-1. **(B)** Effect of G*i* inhibition by PTX on Sp-1 induced by ET-1 in PAECs, as determined by Western blotting. After overnight treatment with 100 ng/mL of PTX, ET-1 (10 nmol/L) was added to PAECs, and after 30 min, western blotting of Sp-1 was performed. Lane 1, PAECs alone (control); lane 2, PAECs + ET-1; lane 3, PAECs + ET-1 + PTX; and lane 4, PAECs + PTX. **(C)** Effect of RhoA inhibition by C3T on Sp-1 induced by ET-1 in PAECs, as determined by Western blotting. After overnight treatment with 0.25 μg/mL of C3T, ET-1 (10 nmol/L) was added to PAECs, and after 30 min, Western blotting of Sp-1 was performed. Lane 1, PAECs alone (control); lane 2, PAECs + ET-1; lane 3, PAECs + ET-1 + C3T 0.25 μg/mL; and lane 4, PAECs + C3T 0.25 μg/mL. **(D)** Effect of the Sp-1 inhibition by mithramycin A on ACVRL-1 induced by ET-1 in PAECs, as determined by western blotting. PAECs were pre-treated with mithramycin A (1 μmol/L) for 30 min before adding ET-1. After 18 h of ET-1 stimulation (10 nmol/L), Western blotting of ACVRL-1 was performed. Lane 1, PAECs alone (control); lane 2, PAECs + ET-1; lane 3, PAECs + ET-1 + mithramycin A; and lane 4, PAECs + mithramycin A. Quantitative results of ACVRL-1 were normalized by the levels of β-actin. Data are expressed as mean ± SE of three to five experiments. Representative immunoblots are shown at the top. *P < 0.05 vs. lane 1, ^†^*P* < 0.05 vs. lane 2.

We investigated the role of Sp-1, which is one of the transcriptional factors for ACVRL-1, in ET-1-stimulated PAECs. Figure 5A shows that, after adding ET-1, the level of Sp-1 was increased within 10 min, and peaked after 15 min. Both G*i* inhibition by PTX and RhoA inhibition by C3T prevented the increase of Sp-1 level in response to ET-1 (Figures 5B,C). These data indicate that rapid RhoA activation *via* G*i* affects Sp-1 regulation. Furthermore, inhibition of Sp-1 by mithramycin A suppressed ET-1-induced ACVRL-1 upregulation (Figure 5D).

## Discussion

**Figure 6:**
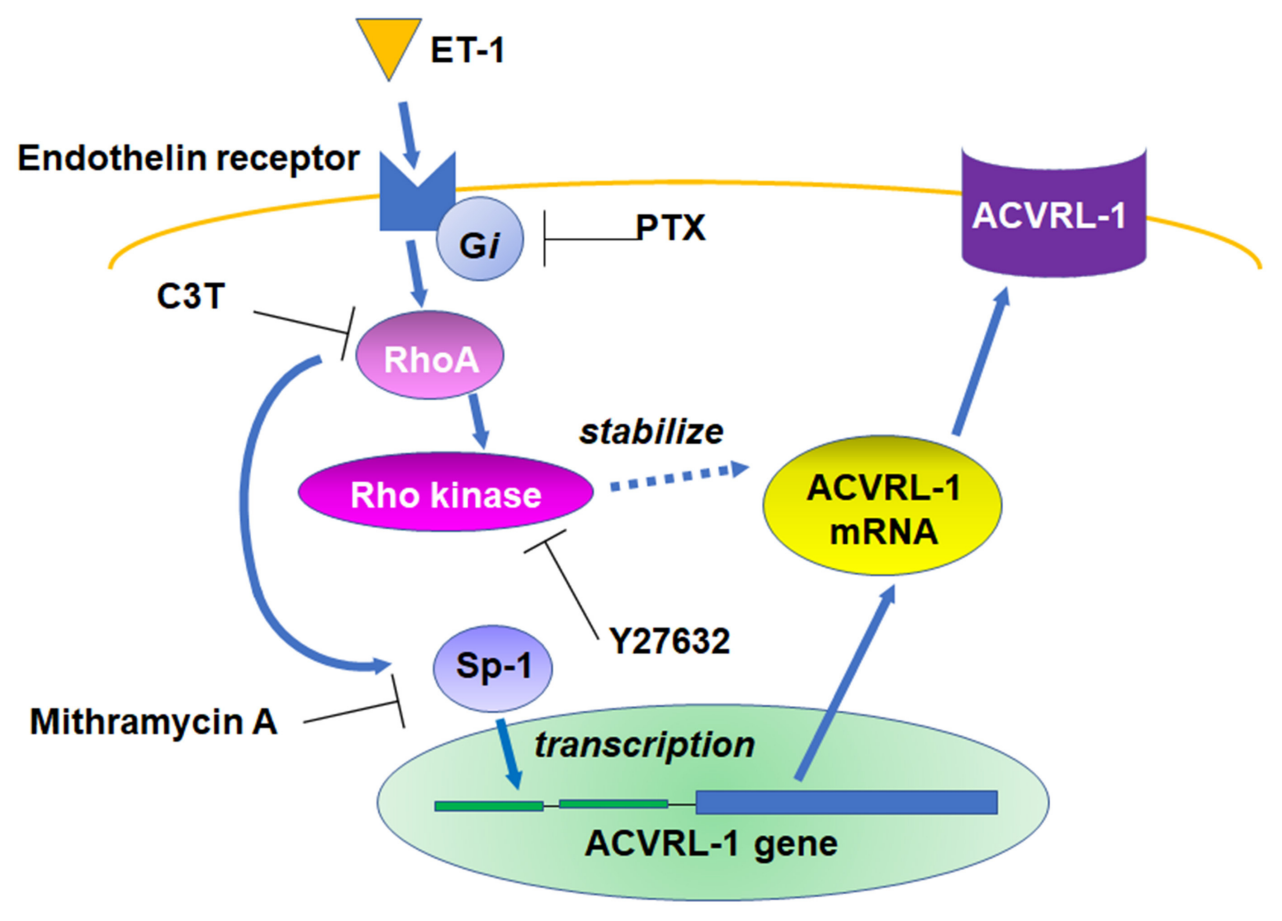
Schema of the signaling pathways leading to ACVRL-1 expression in pulmonary artery endothelial cells in response to ET-1.

We, for the first time, demonstrated that ET-1 upregulated ACVRL-1 expression *via* the Gi/RhoA/Rho kinase pathway in PEACs (Figure 6). Our results suggest that the activation of G*i* and RhoA is associated with the promoter activity of ACVRL-1 *via* Sp-1 and the stability of ACVRL-1 mRNA.

In patients with idiopathic PAH, an ET-1 plasma level is correlated with mean pulmonary arterial pressure and pulmonary vascular resistance (19). It has also been reported that ACVRL-1 expression is increased in the PAECs of patients with idiopathic PAH (20). Taking these clinical findings and our *in vitro* data together, it is highly possible that elevated ET-1 levels actually increase ACVRL-1 expression in PAH patients. In addition, a previous study has shown that ACVRL-1 deficiency induced the synthesis and release of ET-1 (21). This phenomenon might have been caused to maintain the levels of ACVRL-1 expression by increasing ET-1 stimulation.

However, in mice models, there have been opposing results of the effect of TGF-β receptor deficiency on PAH. While Jerkic et al. reported that adult ACVRL-1 heterozygous mice spontaneously developed PAH (22), Gore et al. demonstrated that hypoxia-induced pulmonary hypertension was ameliorated by a deficiency of endoglin, which is a TGF-β receptor. Similar to ACVRL-1, gene mutation in endoglin is implicated in PAH (20). Therefore, the significance of increases in ACVRL-1 due to ET-1 in PAH patients remains unclear and needs to be clarified in future studies. However, since some effects of ET-1 on endothelial cells are favorable to PAH patients, such as ET-B receptor-mediated vasodilation (23), it can be considered that the increased expression of ACVRL-1 by ET-1 is also one of the positive effects for PAH. ACVRL-1 signaling is functionally attenuated in PAH, and thus the increase in ACVRL-1 by ET-1 may work as a compensatory mechanism.

Previous studies have shown that whether the modification of RNA expression by RhoA is upregulated or downregulated depends on the type of molecule (24, 25). Marshall et al. demonstrated that ET-1 upregulated RNA expression of some molecules (e.g., Abra, Srf, and Egr2), which were inhibited by C3T in rat cardiomyocytes (24). On the other hand, Laufs et al. reported that RhoA activation downregulated the expression of endothelial nitric oxide synthase (eNOS) at the post-transcriptional level in endothelial cells. They showed that actin stress fiber reorganization by RhoA activation was associated with the post-transcriptional regulation of eNOSmRNA (25).

Murthy et al. reported that Rac1, which is a small GTP protein similar to RhoA, stabilized Sp-1 expression in macrophages (26). Interestingly, in the current study, ET-1-triggered RhoA activation increased the Sp-1 protein within 15 min, whereas the stabilization of Sp-1 by Rac1 was shown to take several hours (25).

Several studies have reported on the association between ET-1 and the PTX-sensitive G protein (27, 28). Since endothelial cells predominantly express ET-B receptors which are reported to be associated with G*i* (23, 29), we hypothesized that ET-1-induced ACVRL-1 upregulation was mediated *via* an ET-B receptor. However, further mechanisms of correlation between the ET-1/G*i* axis and ACVRL-1 need to be elucidated.

## Conclusion

We for the first time demonstrated that ET-1 upregulated ACVRL-1 expression in human PAECs. G*i*, RhoA, Rho kinase, and Sp-1 are important as the underlying mechanisms of ET-1-induced expression of ACVRL-1 in PAECs.

## Data Availability Statement

The original contributions generated in the study are included in the article/supplementary material, further inquiries can be directed to the corresponding author.

## Author Contributions

KS and YT: conception, design of the work, and manuscript writing. KS and TY: acquisition of data. KS, TM, SY, TK, and AY: analysis and interpretation of data. KN and YT: review and editing. All authors have read and approved the final manuscript.

## Conflict of Interest

KS and TY belong to a department supported by Actelion Pharmaceuticals Japan. This company was not associated with the contents of this study. The remaining authors declare that the research was conducted in the absence of any commercial or financial relationships that could be construed as a potential conflict of interest.
